# A Conserved Gcn2-Gcn4 Axis Links Methionine Utilization and the Oxidative Stress Response in *Cryptococcus neoformans*

**DOI:** 10.3389/ffunb.2021.640678

**Published:** 2021-03-22

**Authors:** Anna K. Stovall, Corey M. Knowles, Murat C. Kalem, John C. Panepinto

**Affiliations:** Department of Microbiology and Immunology, Witebsky Center for Microbial Pathogenesis and Immunology, Jacobs School of Medicine and Biomedical Sciences, University at Buffalo, State University of New York (SUNY), Buffalo, NY, United States

**Keywords:** *cryptococcus neoformans*, fungal pathogen, nitrogen metabolism, methionine, oxidative stress, transcription factor, translation regulation

## Abstract

The fungal pathogen *Cryptococcus neoformans* relies on post-transcriptional mechanisms of gene regulation to adapt to stressors it encounters in the human host, such as oxidative stress and nutrient limitation. The kinase Gcn2 regulates translation in response to stress by phosphorylating the initiation factor eIF2, and it is a crucial factor in withstanding oxidative stress in *C. neoformans*, and amino acid limitation in many fungal species. However, little is known about the role of Gcn2 in nitrogen limitation in *C. neoformans*. In this study, we demonstrate that Gcn2 is required for *C. neoformans* to utilize methionine as a source of nitrogen, and that the presence of methionine as a sole nitrogen source induces eIF2 phosphorylation. The stress imposed by methionine leads to an oxidative stress response at both the levels of transcription and translation, as seen through polysome profiling as well as increased abundance of select oxidative stress response transcripts. The transcription factor Gcn4 is also required for methionine utilization and oxidative stress resistance, and RT-qPCR data suggests that it regulates expression of certain transcripts in response to oxidative stress. The results of this study suggest a connection between nitrogen metabolism and oxidative stress in *C. neoformans* that is mediated by Gcn4, possibly indicating the presence of a compound stress response in this clinically important fungal pathogen.

## Introduction

*Cryptococcus neoformans* is a basidiomycetous fungus that is found ubiquitously in the environment and is also an opportunistic pathogen in humans. As a pathogen, *C. neoformans* is a causative agent of cryptococcosis and is responsible for up to 250,000 deaths annually (Rajasingham et al., 2017). Immunocompromised individuals such as those living with HIV/AIDs and with organ transplants are particularly susceptible. Although advances have been made in antiretroviral therapy and other health interventions, *C. neoformans* infection is still prevalent in developing regions of the world with limited resources and access to healthcare. In addition, growing resistance to current antifungal drugs highlights the need for the development of new therapeutics to treat cryptococcal infections (Smith et al., 2015; Rajasingham et al., 2017; Zafar et al., 2019).

*C. neoformans* enters the human body through inhalation and from there travels to the lungs. Inside the human body, *C. neoformans* encounters stressors that it must adapt to in order to survive: elevated host temperatures, oxidative stress, pH changes, and limitation of nutrients such as glucose and nitrogen (Brown et al., 2007; Esher et al., 2018). Our lab has demonstrated that stress adaptation is dependent on post-transcriptional mechanisms of gene expression regulation, such as mRNA decay and translation regulation. The role of the deadenylase Ccr4 in regulating mRNA decay in response to host temperature has been well-established (Bloom and Panepinto, 2014; Banerjee et al., 2016; Bloom et al., 2017, 2019). Recently, we have begun to characterize the role of the kinase Gcn2 in the response to oxidative stress. Gcn2 phosphorylates the eukaryotic translation initiation factor eIF2, which forms a ternary complex with the initiating Met-tRNA and the 40S ribosomal subunit which scans the transcript for a start codon to begin translation. Phosphorylating eIF2 prevents formation of the ternary complex and inhibits cap-dependent translation in eukaryotic cells (Baird and Wek, 2012; Anda et al., 2017). In *C. neoformans*, exposure to hydrogen peroxide results in phosphorylation of eIF2, global repression of translation and protein synthesis, and promotes expression of genes required for oxidative stress responses. Use of a Gcn2-deficient strain demonstrated that eIF2 phosphorylation is essential to surviving oxidative stress (Leipheimer et al., 2019a). It has yet to be determined whether or not Gcn2 and eIF2 phosphorylation are required for adaptation to other stressors in *C. neoformans*.

One of the most well-studied roles of Gcn2 is its regulation of general amino acid control (GAAC) and translation of the transcription factor Gcn4 in *Saccharomyces cerevisiae*. During amino acid starvation, uncharged tRNAs accumulate and stimulate Gcn2 to phosphorylate eIF2. Increased eIF2 phosphorylation then promotes increased translation of Gcn4, which controls transcription of genes involved in amino acid biosynthesis (Dever et al., 1992; Hinnebusch, 2005). Gcn2's role in regulating amino acid metabolism is conserved broadly throughout eukaryotes (Ye et al., 2015), yet it has not been extensively studied in *C. neoformans*.

Nitrogen is an element that is essential for all living organisms, as it is required for nucleic acids, proteins, vitamins, and other molecules. Throughout the human body, nitrogen can be found in different compounds in different tissues and sera: some examples include urea, ammonia, and free amino acids (Ries et al., 2018). Fungi can occupy different niches of the host either as commensals or as pathogens, including the lungs, phagosomes of phagocytic cells, the cerebrospinal fluid, and the brain. The acquisition and metabolism of nitrogen is an essential aspect of virulence for fungal pathogens such as *Candida albicans* and *Aspergillus fumigatus;* these species will prioritize assimilation of more preferred nitrogen sources such as ammonia or certain amino acids, and then derepress the expression of catabolic genes in order to utilize less-preferred sources in a process known as nitrogen catabolite repression (Ene et al., 2014). Nitrogen catabolite repression has also been described in *C. neoformans* (Kmetzsch et al., 2011; Lee et al., 2011, 2012). However, little is known about the regulatory mechanisms that allow *C. neoformans* to rapidly adapt to different nitrogen conditions in the host.

In this study, we investigated what role eIF2 phosphorylation may play in regulating nitrogen metabolism in *C. neoformans*. We found that Gcn2 is required for *C. neoformans* to grow when methionine is the only available nitrogen source, and that methionine induces eIF2 phosphorylation and represses cap-dependent translation. Ascorbic acid rescued some growth of the *gcn2*Δ strain on methionine and alleviates eIF2 phosphorylation, suggesting a connection between methionine and oxidative stress. We also observed an increased abundance of certain ROS response transcripts in the presence of methionine. In addition, we show that Gcn4, a transcription factor necessary for amino acid metabolism and oxidative stress responses, is linked to the response to methionine-induced oxidative stress. Our results indicate a connection between nitrogen metabolism and oxidative stress, suggesting a model of adaptation where the response to one stressor influences the response to another.

## Materials and Methods

### Cells, Media, and Treatments

All strains of *C. neoformans* used are in the H99 background. The deletion mutant for Gcn2 (CNAG_06174) *gcn2*Δ and *gcn2*Δ:GCN2 strains were reported previously (Leipheimer et al., 2019a). The putative *gcn4*Δ CNAG_06246 knockout strain is from the Madhani deletion collection and was obtained through the Fungal Genetics Stock Center at the University of Kansas. The *gcn4*Δ:*GCN4* strain was generated by cloning the *GCN4* locus into the pBluescript SK II (–) vector carrying the neomycin resistance cassette by *Kpn* I digestion. The vector was inserted into *gcn4*Δ cells using biolistic transformation, and neomycin resistance was used to select for transformants. Transformants were then confirmed via PCR. Sequences for the primers used to amplify and confirm the *GCN4* locus can be found in Supplementary Table 1.

Starter cultures of cells were grown in 5-6 mL of yeast extract-peptone-dextrose (YPD) media (Difco DF0428) in 14 mL snap-cap tubes and incubated at 30°C, shaking, for ~16 h. For growth to mid-log phase, cells were seeded at OD 0.15–0.2 in baffled flasks. Media used in experiments include Yeast Nitrogen Base without added ammonium or amino acids (YNB) (Difco DF0919) with 2% dextrose (YNB-Dex), supplemented with 10 mM of either ammonium sulfate or methionine (Referred to as ammonium media or methionine media, respectively.)

Antioxidants used in this study include N-acetylcysteine (Sigma-Aldrich A7260), ascorbic acid (Sigma-Aldrich A92902), and EUK-134 (Sigma-Aldrich SML0743). N-acetylcysteine and ascorbic acid were both used at 10 mM, and EUK-134 was used at 200 μM.

### Serial Dilution Plates

To assess growth of different *C. neoformans* strains on different nitrogen sources and antioxidant conditions, cells were spotted onto agar plates in a serial dilution assay. Overnight cultures were washed twice and resuspended to an OD_600_ of 1.0 in sterile deionized water. The resuspension was then diluted 10-fold five times in water. 5 μL of each dilution were spotted onto agar plates supplemented with YNB-Dex and a single nitrogen source, then incubated for 2 days at 30°C. Images were captured after 2 days of incubation. Nitrogen sources include ammonium sulfate (Fisher Scientific A702-500), L-methionine (Sigma-Aldrich M9625-25G), urea (Fisher Scientific U15-500), L-glutamine (VWR 0374-500G), L-glycine (Fisher Scientific G46-500), L-asparagine (Fisher Scientific BP373-100), L-arginine (Sigma-Aldrich 11009-25G-F), and L-proline (Sigma-Aldrich P0380-100G). Hydrogen peroxide (Fisher Scientific H323) was added to induce oxidative stress, and sodium nitrite (Fisher Scientific S347) was added to induce nitrosative stress.

### Plate Reader Assay

Overnight cultures of wild-type, *gcn2*Δ, and *gcn2*Δ:GCN2 strains were washed and resuspended to an OD_600_ of 0.3 in sterile deionized water. 100 μL of resuspended cells were added to 100 μL of media per well in a 96-well plate. Plates were incubated in a BioTek Synergy H1 Hybrid Reader for 48 h at 30°C with double orbital shaking, measuring OD_600_ in each well every 15 min. Changes in OD_600_ were graphed using GraphPad Prism software.

### Time Course and Protein Isolation for Western Blotting

Overnight cultures of wild-type cells were grown to mid-log phase (OD_600_ 0.5-0.6) in YNB-dextrose with 10 mM ammonium sulfate, shaking, at 30°C. Cells were washed and resuspended in fresh ammonium media or methionine media, and incubated an additional 3 h at 30°C with shaking. During the 3-h incubation 5-6 mL aliquots of cells were collected, pelleted, and flash frozen.

For protein isolation, cell pellets were washed with water at 4°C and resuspended in protein lysis buffer (15 mM HEPES pH 7.4, 10 mM KCl, 5 mM MgCl_2_) with added protease/phosphatase inhibitor cocktail and DTT. Cells were lysed with mechanical disruption via bead beating. Lysates were cleared using centrifugation at 15,000 rpm for 10 min at 4°C. Total protein in each lysate was quantified using the Pierce BCA Protein Assay kit (ThermoFisher catalog no. 23225).

### Western Blot

Western blot analysis was performed as previously described (Leipheimer et al., 2019a). Briefly, 20 μg total protein were run on BioRad Mini-Protean TGX stain-free gels (BioRad 4568084) at 200V for 40 min and gels were imaged to capture images of total protein in each lane using a BioRad GelDoc XR+ Imager and ImageLab software. Protein was transferred to nitrocellulose membranes using a BioRad Trans-Blot Turbo System and the Trans-Blot Turbo kit (BioRad 170-4270RTA). Blots were then blocked in Odyssey Blocking Buffer (Licor 927-60001) and incubated with a rabbit primary antibody against EIF2S1 (phospho-S51) (Abcam ab32157) at a concentration of 1:1000 in blocking buffer and TBST, followed by incubation with an IR680-conjugated secondary antibody (Licor 926-68023) at a concentration of 1:10,000 in blocking buffer and TBST. Blots were imaged using the LiCOR Odyssey Imaging System.

### Polysome Profiling

Overnight cultures of wild-type and *gcn2*Δ cells were grown to mid-log phase (OD_600_ = 0.5-0.6) in YNB-dextrose with 10 mM ammonium sulfate, shaking, at 30°C. Cells were washed and resuspended in fresh ammonium media, methionine media, or methionine media supplemented with 10 mM ascorbic acid and incubated an additional 60 min at 30°C with shaking. Cycloheximide (Acros Organic 66-81-9) was added to cultures at a concentration of 0.1 mg/mL before cells were pelleted and flash frozen. Cell pellets were washed and resuspended in polysome lysis buffer (20 mM Tris-HCl [pH 8], 2.5 mM MgCl, 200 mM KCl, 1% Triton X-100, 0.1 mg/ml cycloheximide) prior to lysis via bead beating. An additional 300 μL of lysis buffer was added to lysates, and lysates were cleared via centrifugation at 15,000 rpm for 5 min at 4°C.

Polysome profiling was performed as described previously (Leipheimer et al., 2019a). Briefly, 250 μg of total RNA from each sample was loaded onto a 10–50% sucrose gradient and subjected to ultracentrifugation at 39,000 rpm for 2 h at 4°C in an SW-41 rotor. Gradients were then run through a flow cell measuring absorbance at 254 nm using a UA-6 UV-visible detector, and absorption data was recorded using DataQ software.

### Flow Cytometry and Statistical Analysis

Overnight cultures of wild-type and *gcn2*Δ cells were grown to mid-log phase (OD_600_ = 0.5-0.6) in ammonium media, shaking, at 30°C. Cells were then washed and resuspended in 1mL of one of the following: fresh ammonium media, ammonium media supplemented with 2 mM hydrogen peroxide as a positive control, methionine media, or methionine media supplemented with 10 mM ascorbic acid. 1 mL suspensions of cells were incubated for 60 min, with mid-log culture cells used for Time 0, and CellRox Deep Red Dye (ThermoFisher C10491) was added to each culture during the final 30 min of incubation. Following staining with CellRox Deep Red dye, cells were washed once and resuspended in PBS. Fluorescent signal was analyzed with a BD LSR Fortessa flow cytometer using the APC channel (640 nm), and 100,000 cells per sample were analyzed. Data analysis was conducted using FlowJo v10.0 software, calculating percentage of cells positive for CellRox in each population along with the mean fluorescence intensities of each CellRox+ population. Statistical analysis was performed using a Kruskal-Wallis test with *post-hoc* analysis performed using Dunn's test.

### RNA Time Course

Overnight cultures of wild-type, *gcn2*Δ, or *gcn4*Δ cells were grown to mid-log phase (OD_600_ = 0.5-0.6) in ammonium media, shaking, at 30°C. Cells were washed and resuspended in fresh ammonium media or methionine media, and incubated an additional 2 h at 30°C with shaking. During the 2-h incubation, 5-6 mL aliquots of cells were collected, pelleted, and 50 μL of Buffer RLT (Qiagen 79216) with 2-mercaptoethanol was added to pellets prior to flash freezing. Pellets were stored at −80°C.

### RNA Isolation, Preparation, qRT-PCR, and Statistical Analysis

Frozen cell pellets were lysed with mechanical disruption via bead beating. Total RNA was isolated using the QIAGEN RNEasy Mini Kit (QIAGEN 74106) according to manufacturer's instructions and resuspended in RNAse-free water. After isolation, total RNA samples were DNAse-treated with Invitrogen TURBO DNA-free Kit (ThermoFisher Scientific AM1907) to remove genomic DNA from samples. The Applied Biosystems High-Capacity cDNA Reverse Transcription Kit (ThermoFisher Scientific 4368813) was then used to synthesize cDNA from 750 ng of total RNA template using random hexamers.

For qPCR, standards were generated by diluting cDNA samples four-fold, five times. Samples assayed for mRNA abundance were diluted 1:5 in water. 1 μL of diluted cDNA was added to 5 μL of 2x SYBR Green Blue Mix (PCR Biosystems) and 4 μL of primers, for a 10 μL total reaction volume. Reactions were run using a Bio-Rad CFX Connect Real-Time PCR Detection System with the following cycle conditions: denaturation at 95°C for 5 min; 40 amplification cycles at 95°C for 10 s and 60°C for 30 s; and melting curve analysis at a gradient from 60°C to 100°C. *CHS6* was used as a housekeeping gene. Primer sequences used can be found in Supplementary Table 1.

For qPCR data analysis, a standard curve was created using the Ct values for each four-fold dilution standard, a linear trendline was generated to fit the curve, and the equation of the trendline was used to calculate abundance of each transcript. Abundance values for each examined transcript were normalized to *CHS6* abundance at the same timepoint in the same strain and condition. Normalized values were graphed as fold change from the time 0 abundance, with error bars representing standard error of the mean. Statistical analysis was performed using one-way ANOVA, comparing timepoints within each strain and condition as well as strains and conditions at each timepoint, with *post-hoc* analysis performed through Sidak's multiple comparisons test.

## Results

### *C. neoformans* Cells Deficient in Gcn2 Show Diminished Growth in the Presence of Methionine

**Figure 1:**
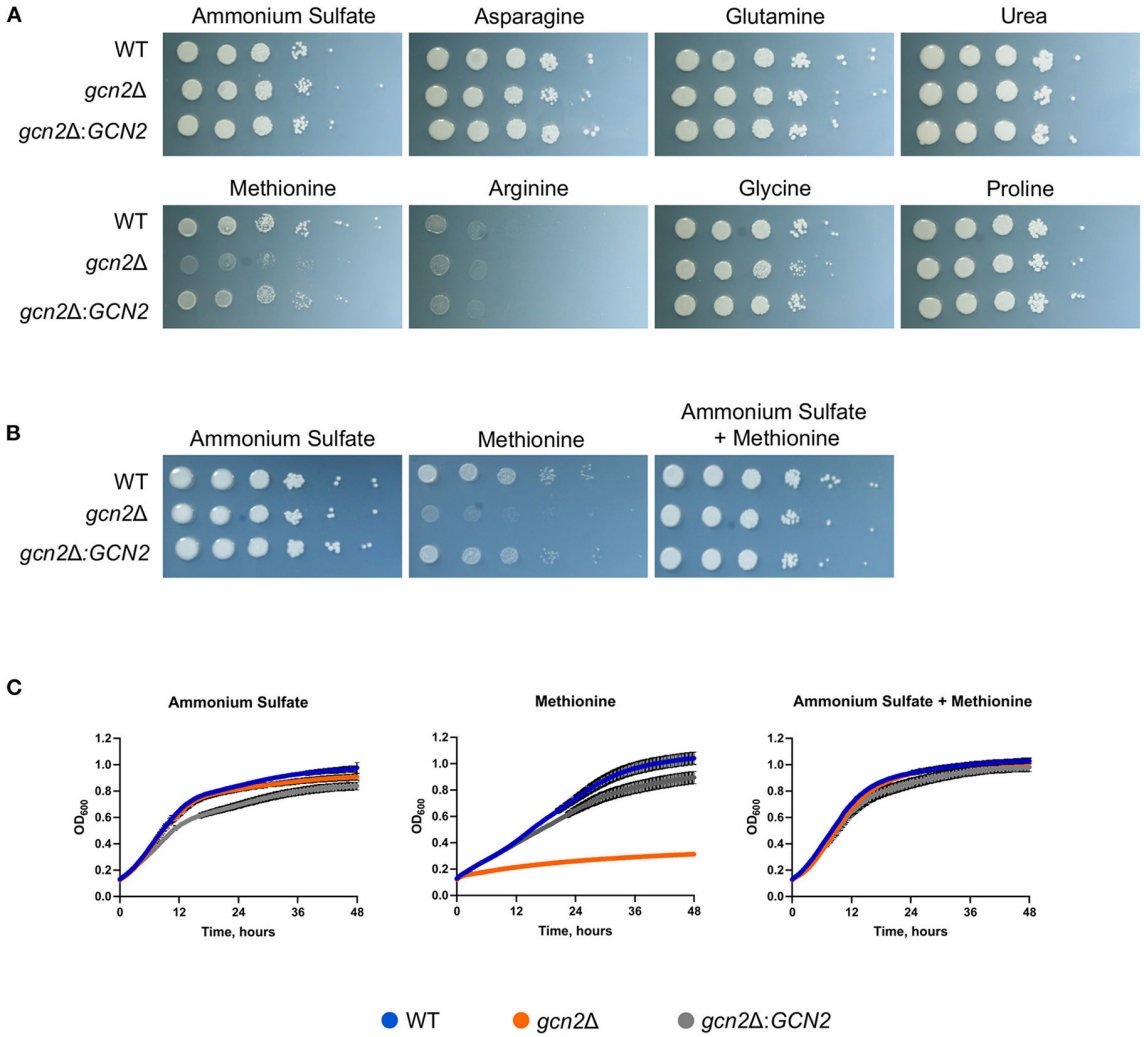
Gcn2 is required for utilization of methionine as a nitrogen source. **(A)** Serial dilution analysis of wild-type *C. neoformans* alongside *gcn2*Δ and its complement *gcn2*Δ:*GCN2*. Cells were spotted on YNB agar plates supplemented with 2% dextrose and 10 mM of the indicated compound as the sole nitrogen source. Plates were incubated at 30° for 2 days before imaging. Images shown are representative of four biological replicates. **(B)** Serial dilution analysis of wild-type, *gcn2*Δ and *gcn2*Δ:*GCN2* strains. Cells were spotted on YNB agar plates supplemented with 2% dextrose, 10 mM ammonium sulfate, 10 mM methionine, or 10 mM ammonium + 10 mM methionine. Plates were incubated at 30° for 2 days before imaging. Images shown are representative of three biological replicates. **(C)** Wild-type, *gcn2*Δ and *gcn2*Δ:*GCN2* strains were assayed for growth in liquid media over 48 h at 30° in a plate reader assay. Data shown are from two biological replicates.

We began by determining whether Gcn2 and eIF2 phosphorylation were required to regulate nitrogen metabolism in *C. neoformans*. To explore this possibility, we performed serial dilution assays where wild-type, *gcn2*Δ, and the complement strain *gcn2*Δ*:GCN2* were spotted onto agar containing different amino acids or nitrogen-containing compounds that would be the sole nitrogen sources available to cells. We observed that the *gcn2*Δ strain shows uninhibited growth on ammonium, urea, and most of the amino acids tested. The wild-type strain showed some inhibited growth on arginine, and *gcn2*Δ growth was comparable to wild-type. However, *gcn2*Δ showed severely diminished growth in the presence of methionine compared to wild-type and the complement strain (Figure 1A). The *gcn2*Δ strain's inability to grow on methionine piqued our interest. We speculated that methionine metabolism may have imposed some type of stress on the cell that would require Gcn2 activation. We also considered the possibility that methionine's presence may have facilitated Gcn2 activation through another route, such as signaling through the Gpr4 receptor and activation of the cAMP/PKA pathway (Bahn and Jung, 2013). To assess these possibilities, we spotted cells on plates containing both 10 mM ammonium and 10 mM methionine as nitrogen sources. We observed that when ammonium was available as a nitrogen source, either with or without added methionine, the *gcn2*Δ strain's growth was comparable to that of the wild-type and the complement strain (Figure 1B). The growth phenotypes observed on agar plates were recapitulated in liquid media growth assays (Figure 1C).

Together, these results suggest that the kinase Gcn2 is required to catabolize methionine and use it as a source of nitrogen. The results of these experiments are consistent with what is known about nitrogen catabolite repression in *C. neoformans*: as ammonium is an easily assimilated source of nitrogen, the cell will preferentially utilize it for nitrogen before using any other compounds such as urea or amino acids (Lee et al., 2011). Therefore, when there is ammonium available for cells, they will utilize that before utilizing methionine and undergoing any stress that methionine metabolism might impose which leads to Gcn2 activation.

### Phosphorylation of eIF2 and Global Translation in the Presence of Methionine

**Figure 2:**
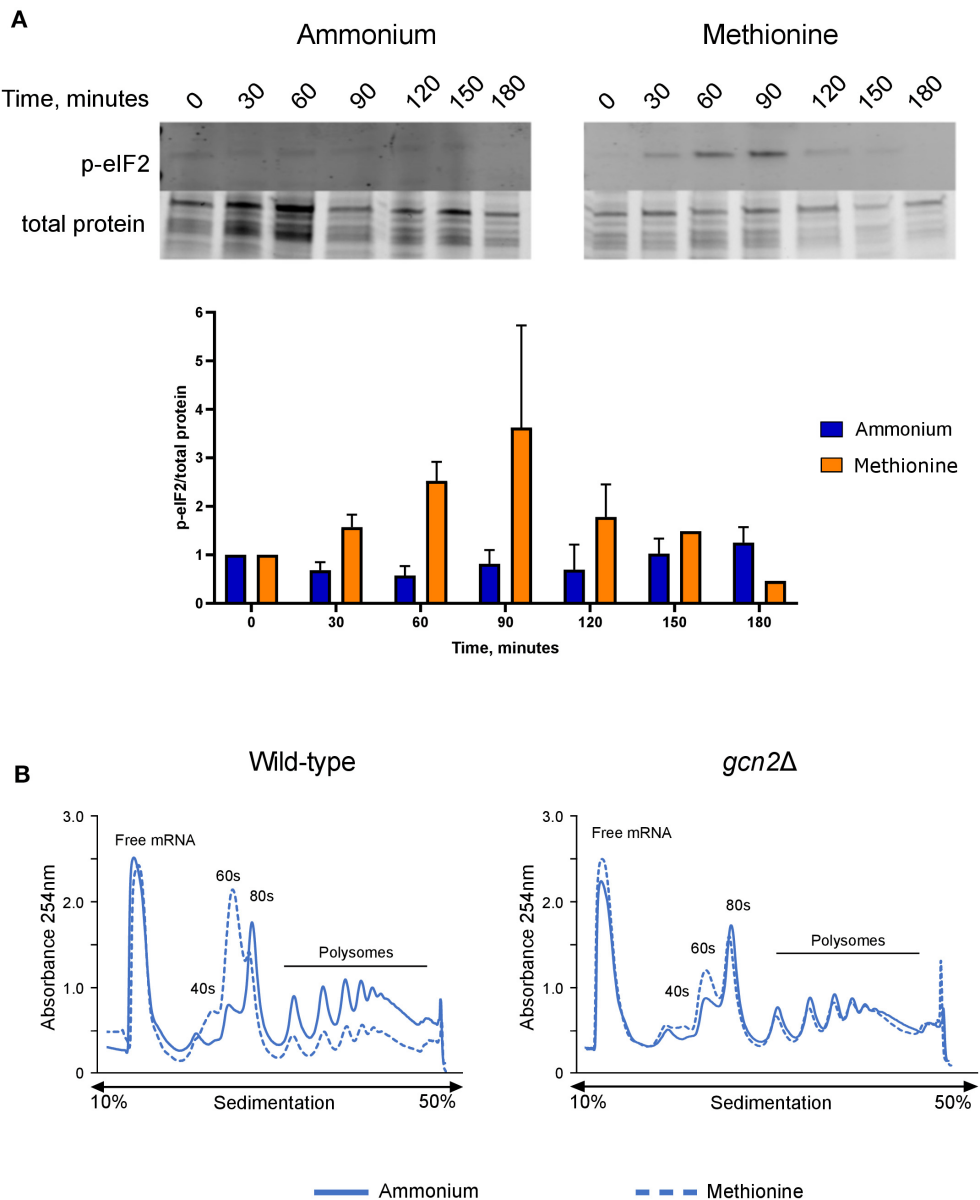
Effects of methionine incubation on eIF2 phosphorylation and translation in *C. neoformans*. **(A)** Western blot analysis of eIF2 phosphorylation. Wild-type cells were grown to mid-log phase before resuspension in fresh minimal defined media containing 10 mM of either ammonium sulfate or methionine. Cells were collected in 30-min increments over a 3-h time course. Protein extracted from cells was analyzed by western blot for phosphorylated eIF2. Images shown are representative of three biological replicates. Densitometry analysis was performed by normalizing p-eIF2 signal to total protein signal, and data shown are the averages of three biological replicates. **(B)** Polysome profiling analysis of wild-type and *gcn2*Δ cells. Mid-log cells were incubated in minimal defined media with ammonium or methionine for 60 min. Cells were then treated with cycloheximide to arrest ribosomes, pelleted, and flash frozen in liquid nitrogen. Cells were lysed, and lysates were loaded onto sucrose gradients. Gradients were ultracentrifuged for 2 h at 39,000 rpm and 4°C to separate the contents of the lysate by density. After spinning, samples were run through a flow cell, where the absorbance of the sample at 254 nm was measured, and polysome profiles were generated. Images shown are representative of two biological replicates.

After observing that *gcn2*Δ cells could not utilize methionine for nitrogen, we hypothesized that the presence of methionine as the sole nitrogen source for *C. neoformans* would lead to Gcn2 activation, resulting in increased levels of eIF2 phosphorylation. We grew wild-type cells in yeast nitrogen base (YNB) media supplemented with 2% dextrose and 10 mM of either ammonium sulfate or methionine over a 3-h time course, collecting cells in 30-min increments. Cells were lysed and analyzed via Western blot for levels of phosphorylated eIF2, which were compared to total protein. In wild-type cells incubated with methionine, levels of p-eIF2 increased during the first half of the time course, with phosphorylation peaking at 60 and 90 min, followed by a sharp decline at 120 min. Comparatively, cells incubated with ammonium maintained a basal level of phosphorylated eIF2 and showed no noticeable change in levels at all time points (Figure 2A). Densitometry analysis of phosphorylated eIF2 relative to total protein revealed quantitative trends that were overall consistent with the western blots, and each biological replicate displayed the same trends of increasing and decreasing p-eIF2 levels, yet the magnitudes differed between replicates which confounded our attempts to perform statistical analyses on the densitometry.

We then used polysome profiling to look at the effects of methionine utilization on global translation in the cell. After incubating wild-type cells in methionine media for 60 min, we observed a loss of polysomes, suggesting translational repression. In addition, we also observed a reduction in the 80S monosome peak and a concomitant increase in the 60S subunit peak, which indicates a down-regulation of translation initiation. However, *gcn2*Δ cells grown in methionine did not exhibit the repression of polysome peaks, reduction in monosome peak and increase in 60S peak seen in the wild-type cells and more closely resembled the *gcn2*Δ cells grown in ammonium (Figure 2B). Together, these results suggest that the translation repression observed in methionine-grown wild-type cells are a result of eIF2 phosphorylation by Gcn2 and involves an inhibition of translation initiation.

### Methionine and Oxidative Stress

Deletion of *GCN2* in *C. neoformans* results in sensitivity to oxidative stress (Leipheimer et al., 2019a). Incubating WT cells in H_2_O_2_ induced robust eIF2 phosphorylation, and the *gcn2*Δ mutant grows poorly in the presence of H_2_O_2_, indicating that eIF2 phosphorylation is an essential component of the oxidative stress response. Methionine metabolism is tied to several cellular processes in eukaryotes such as signaling, proliferation, and S-adenosylmethionine synthesis. In addition, methionine contributes to glutathione synthesis and antioxidant defenses (Walvekar and Laxman, 2019). Thus, we considered it possible that methionine metabolism could be tied to the oxidative stress response via eIF2 phosphorylation.

**Figure 3:**
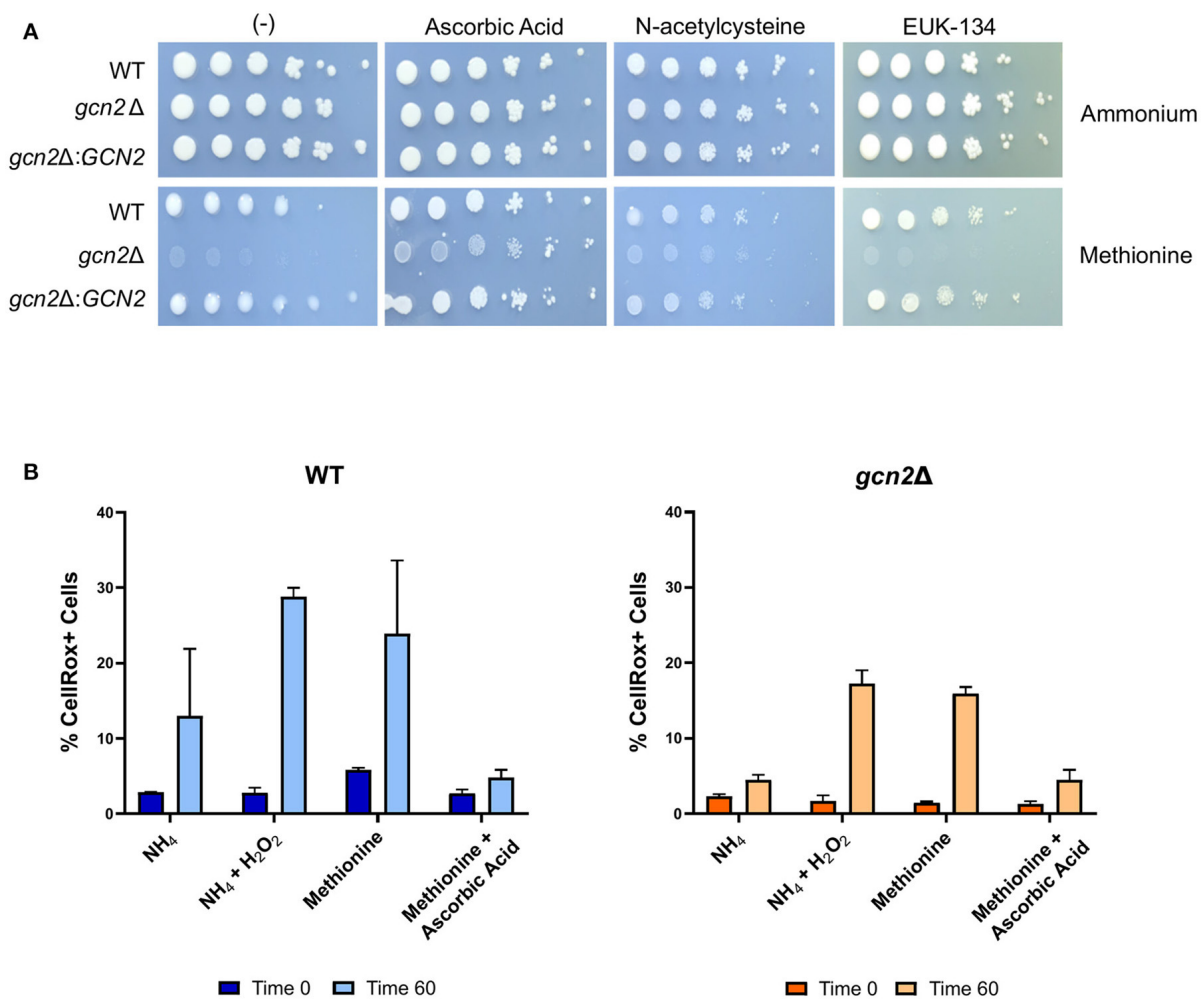
Growth in methionine is linked to induction of oxidative stress. **(A)** Serial dilution analysis of cells in the presence of different nitrogen sources and antioxidants. Wildtype, *gcn2*Δ, and *gcn2*Δ:*GCN2* serial dilutions were spotted onto agar plates containing YNB, 2% dextrose, 10 mM of either ammonium sulfate or methionine, and one of the following antioxidants: 10 mM ascorbic acid, 10 mM N-acetylcysteine, or 200 μM of EUK-134. Plates were incubated at 30° for 2 days before imaging. Image shown are representative of three biological replicates. **(B)** Flow cytometry analysis of cells incubated in ammonium or methionine media supplemented with 2 mM hydrogen peroxide or 10 mM ascorbic acid, respectively. CellRox Deep Red Dye was used to detect intracellular ROS accumulation after 0 or 60 min of incubation. Data presented are the averages of two biological replicates.

We asked if the reduced growth of *gcn2*Δ on methionine was tied to its sensitivity to reactive oxygen species (ROS), and we hypothesized that the addition of antioxidants to growth media would rescue growth of *gcn2*Δ in the presence of methionine. We tested the following antioxidants: N-acetylcysteine, a synthetic precursor of cysteine and glutathione (Sun, 2010; Aldini et al., 2018); ascorbic acid, which is a scavenger of superoxide, singlet oxygen, hydrogen peroxide, and hydroxyl radicals (Beyer, 1994; Guaiquil et al., 2001); and EUK-134, a synthetic superoxide dismutase (Giles et al., 2005). When we supplemented ammonium or methionine plates with 10 mM N-acetylcysteine or 200 μM EUK-134, we saw no change in *gcn2*Δ growth compared to plates lacking antioxidants. However, we observed an increase in *gcn2*Δ growth on methionine plates supplemented with 10 mM ascorbic acid. Together, these results show that ascorbic acid, but not N-acetylcysteine or EUK-134, rescued growth of Gcn2-deficient cells on methionine agar (Figure 3A). The fact that ascorbic acid is the only antioxidant to rescue growth could suggest accumulation of a specific type of reactive oxygen species in the presence of methionine. The inability of N-acetylcysteine to rescue growth suggests that there is no defect in glutathione synthesis or redox balance in *gcn2*Δ cells. The inability of EUK-134 to rescue *gcn2*Δ growth suggests that superoxide is not the predominant reactive oxygen species accumulating in cells. Thus, if the methionine growth defect is due to ROS accumulation, it is likely that either hydrogen peroxide or hydroxyl radicals are the predominant ROS present in *gcn2*Δ cells.

We then utilized flow cytometry to measure the accumulation of intracellular ROS in wild-type and *gcn2*Δ cells incubated in ammonium or methionine supplemented with either hydrogen peroxide or ascorbic acid, respectively. CellRox Deep Red Dye was used to detect intracellular ROS. After 60 min of incubation with methionine, we observed a modest increase in CellRox+ cells in both wild-type and *gcn2*Δ cells incubated in methionine, suggesting increasing ROS accumulation. Lack of statistical significance reinforces our earlier observations that methionine utilization is a mild stressor, and that inability to contend with even the mild ROS stress induced by methionine utilization has severe biological implications on cellular fitness. These results are consistent with the trends of eIF2 phosphorylation observed in cells grown in methionine media (Figure 2), suggesting that this accumulation of ROS is what triggers Gcn2 activation. Peroxide treatment was included as a positive control, and cells treated with peroxide trended to a higher percentage of CellRox+ staining, however, this increase did not meet statistical significance. The addition of ascorbic acid to methionine media prevented the accumulation of ROS in both wild-type and *gcn2*Δ strains (Figure 3B). Despite fluctuations in the total percentages of CellRox+ cells, the mean fluorescent intensities of CellRox+ cells for each group did not change between timepoints (Supplementary Figure 1).

**Figure 4:**
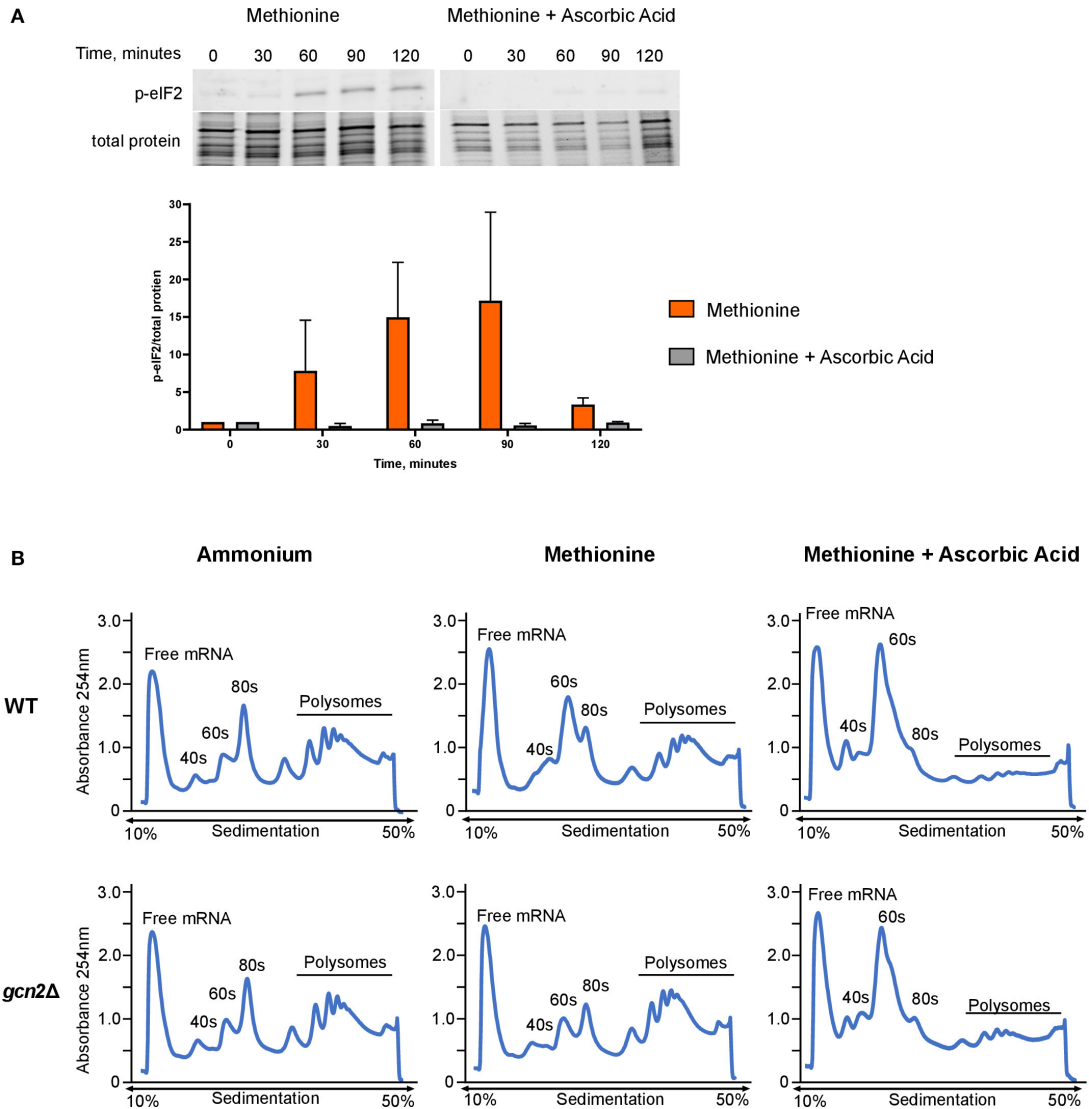
Effects of methionine and ascorbic acid on eIF2 phosphorylation and translation. **(A)** Western blot analysis of eIF2 phosphorylation in cells incubated in methionine media with ascorbic acid. Wild-type cells were grown to mid-log phase before resuspension in fresh minimal defined media containing 10 mM of methionine, with or without the addition of 10 mM ascorbic acid. Cells were collected in 30-min increments over a 3-h time course. Protein extracted from cells was analyzed by western blot for phosphorylated eIF2. Images shown are representative of five biological replicates. Densitometry analysis was performed by normalizing p-eIF2 signal to total protein signal, and data shown are the averages of five biological replicates. **(B)** Mid-log cells were incubated in minimal defined media with ammonium, methionine, or methionine supplemented with 10 mM ascorbic acid for 60 min. Cells were then harvested and processed for polysome profiling as previously described. Images shown are representative of two biological replicates.

Next, we asked whether administering ascorbic acid to cells grown in methionine would reduce eIF2 phosphorylation. To test this, we performed Western blot analysis on wild-type cells grown in methionine media, supplemented with 10 mM ascorbic acid. We observed greatly reduced levels of eIF2 phosphorylation in the ascorbic acid-treated cells compared to the cells grown in methionine without ascorbic acid (Figure 4A). This result further suggests that the presence of methionine as a sole source of nitrogen leads to the induction of an oxidative stress response. As with the results shown in Figure 2A, densitometry analysis of phosphorylated eIF2 relative to total protein revealed consistent trends of increasing and decreasing p-eIF2 levels in each biological replicate, however the magnitude of the increases and decreases differed, confounding our attempts to perform statistical analysis.

As ascorbic acid abolishes methionine-induced eIF2 phosphorylation, we hypothesized that it would alleviate translation repression seen in wild-type cells grown in methionine. We incubated mid-log wild-type and *gcn2*Δ in ammonium or methionine media supplemented with 10 mM of ascorbic acid for 60 min, harvested cells, and performed polysome profiling. Ascorbic acid had no effect on the polysomes of wild-type cells grown in ammonium media (Supplementary Figure 2). However, when cells were incubated in methionine with ascorbic acid, we observed a near-total loss of polysomes. In addition, there was an increase in RNA found in the 40S and 60S subunits (Figure 4B). These results indicate that ascorbic acid represses translation in methionine. As we observed this repression in both wild-type and *gcn2*Δ cells, we concluded that ascorbic acid's effects on translation are independent of eIF2 phosphorylation, but serve to allow translational repression necessary to partially suppress the peroxide sensitivity of the *gcn2*Δ mutant.

### ROS Response Gene Expression

Our results thus far point to an interconnectedness between methionine metabolism and the oxidative stress response in *C. neoformans*. We next asked whether the presence of methionine as a sole source of nitrogen would influence expression of genes involved in oxidative stress responses. We incubated wild-type and *gcn2*Δ cells in either ammonium or methionine media over a 2-h time course, harvesting cells in 30-min increments. Total RNA was isolated and used as a template for cDNA synthesis, and then cDNA was analyzed by qPCR for abundance of *ERG110* (CNAG_05842) which we previously reported to exhibit increased expression in the *gcn2*Δ mutant during peroxide exposure, and glutathione-S-transferase (CNAG_04110) and glutathione peroxidase 1 (CNAG_02503), that are involved in glutathione metabolism and the oxidative stress response in *C. neoformans* (Missall et al., 2005; Chang et al., 2007 Upadhya et al., 2013; Leipheimer et al., 2019a). *CHS6* was also examined as a housekeeping gene. As methionine plays roles in glutathione synthesis and redox balance (Walvekar and Laxman, 2019), we hypothesized that cells incubated in methionine would show upregulation of genes that code for proteins involved in those processes, such as glutathione synthase, glutathione peroxidase, and glutathione-s-transferase. We also expected to determine whether any other genes canonically involved in the *C. neoformans* antioxidant response had their expression regulated by eIF2 phosphorylation induced by methionine exposure.

**Figure 5:**
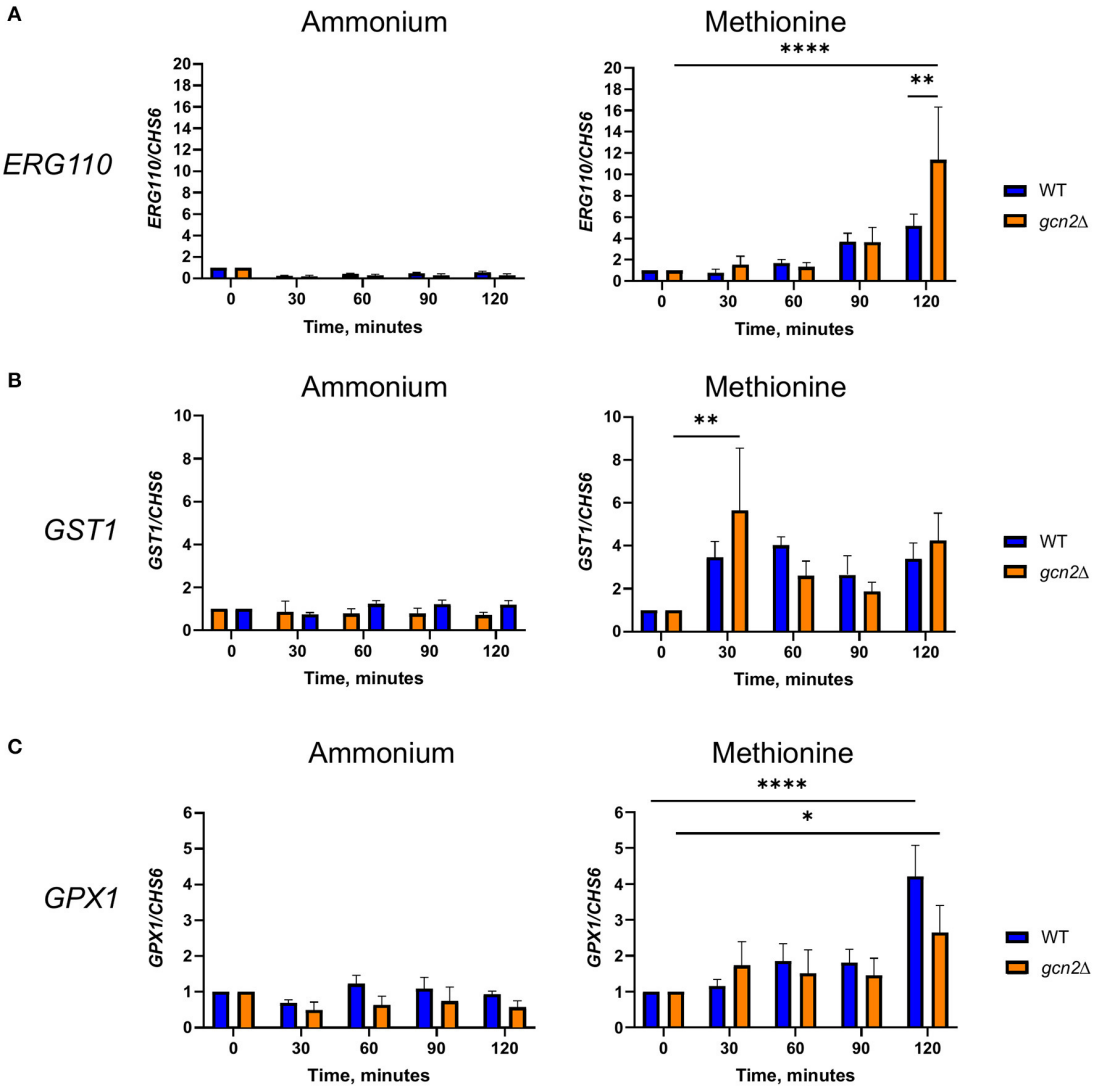
RT-qPCR analysis of steady-state abundance of select oxidative stress response transcripts in methionine. Mid-log wild-type or *gcn2*Δ cells were resuspended in fresh ammonium or methionine media. Cells were collected over a 2-h time course in 30-min increments. Total RNA extracted from cells was used to synthesize cDNA for qPCR analysis of the abundance of *ERG110* **(A)**, *GST1* **(B)**, and *GPX1* **(C)**. Abundance values for each gene were normalized to *CHS6* values in the same strain at the same timepoint. Data shown are from five biological replicates. ^*^p < 0.05, ^**^p < 0.01, ^****^p < 0.0001.

For *ERG110*, we observed no change in steady-state transcript abundance relative to *CHS6* in either wild-type or *gcn2*Δ cells grown in ammonium. However, *ERG110* abundance gradually increased during the time course in cells grown in methionine (*p* < 0.0001) with abundance in *gcn2*Δ being greater than in wild-type at time 120 (*p* < 0.01) (Figure 5A), consistent with what was observed previously during peroxide exposure (Leipheimer et al., 2019a). Abundance of *GPX1* followed a similar pattern, with abundance increasing over time (*p* < 0.05) but without any statistically significant differences between the two strains (Figure 5B). For *GST1*, we observed no changes in abundance in ammonium-grown cells but a rapid increase by the 30-min timepoint in cells grown in methionine in both strains (*p* < 0.01) (Figure 5C). Abundances of other transcripts such as catalase, thiol-specific antioxidase, thioredoxin reductase, and glutathione synthase remained largely unaffected by methionine (Supplementary Figure 3). Together, these results indicate that the presence of methionine as a sole nitrogen source induces an oxidative stress response at the transcriptional level, resulting in upregulation of genes required to address oxidative stress.

### A Putative Gcn4 Ortholog Connects Methionine Utilization and ROS Resistance in *C. neoformans*.

A putative homolog of Gcn4 (CNAG_06246) was identified by Wallace et al. in a recent ribosome profiling study by the presence of obvious ribosome-bound upstream open reading frames in its 5' leader in addition to a BZIP transcription factor domain (Wallace et al., 2020). In addition to its role in amino acid biosynthesis, Gcn4 is also required for oxidative stress responses in yeast, and its expression is regulated by Gcn2 and eIF2 phosphorylation (Hinnebusch, 2005; Duncan et al., 2018). Thus, we were interested in whether Gcn4 was also required for the response to oxidative stress or for amino acid metabolism in *C. neoformans*.

**Figure 6:**
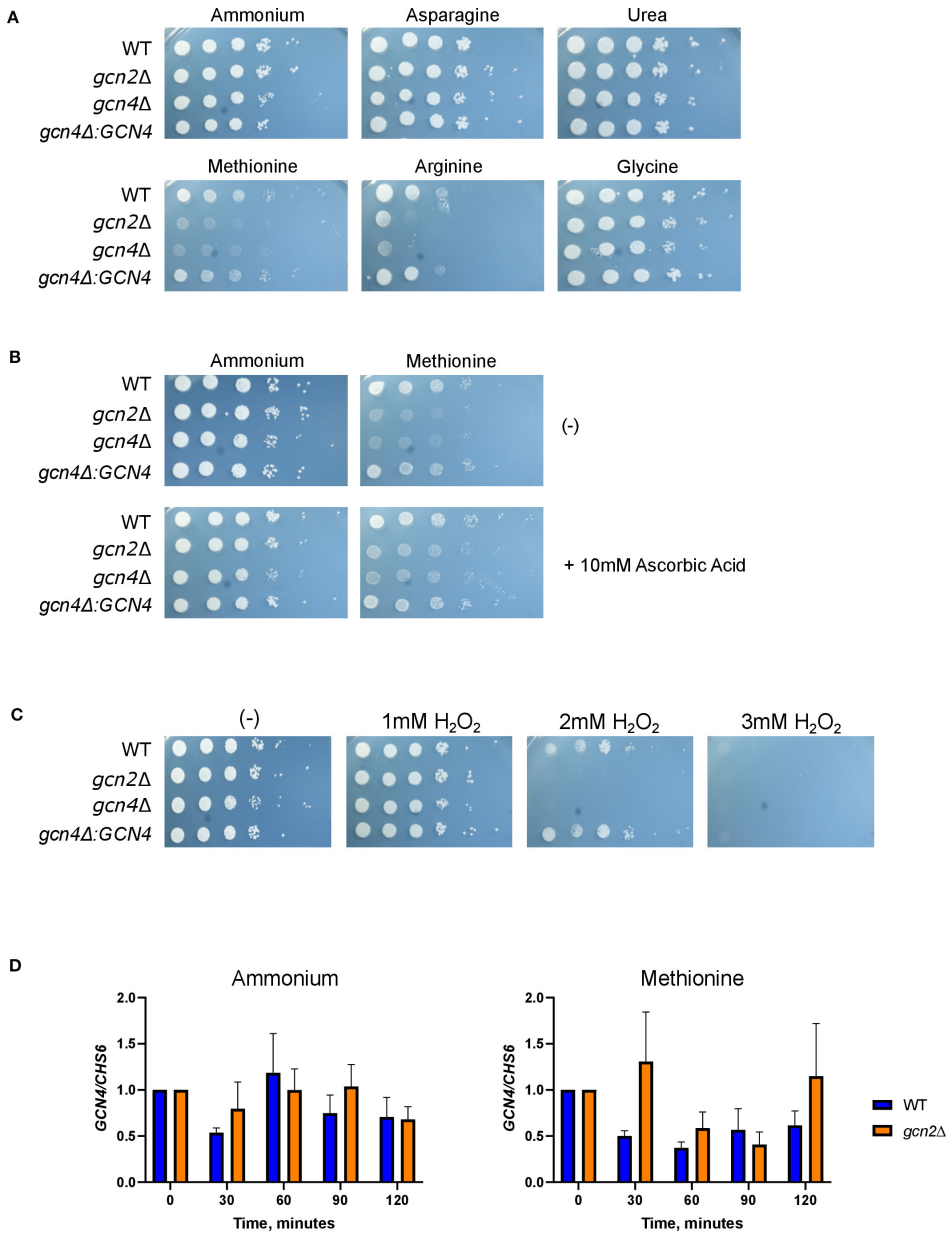
Gcn4 connects methionine metabolism and oxidative stress in *C. neoformans*. **(A)** Serial dilution analysis of wild-type, *gcn2*Δ, and *gcn4*Δ cells. Cells were spotted on YNB agar plates supplemented with 2% dextrose and 10 mM of the indicated compound as the sole nitrogen source. Plates were incubated at 30° for 2 days before imaging. Images shown are representative of three biological replicates. **(B)** Serial dilution plate analysis of antioxidants combined with methionine. Wild-type, *gcn2*Δ, and *gcn4*Δ serial dilutions were spotted onto agar plates containing YNB, 2% dextrose, 10 mM of either ammonium sulfate or methionine, with or without the addition of 10 mM ascorbic acid. Plates were incubated at 30° for 2 days before imaging. Image shown are representative of three biological replicates. **(C)** Serial dilution plate analysis of peroxide sensitivity. Wild-type, *gcn2*Δ, and *gcn4*Δ serial dilutions were spotted onto agar plates containing YNB, 2% dextrose, 10 mM ammonium sulfate, and the indicated concentration of hydrogen peroxide. Plates were incubated at 30° for 2 days before imaging. Images shown are representative of three biological replicates. **(D)** RT-qPCR analysis of steady-state abundance of the *GCN4* transcript in ammonium or methionine. Mid-log wild-type or *gcn2*Δ cells were resuspended in fresh ammonium or methionine media for incubation during a 2-h time course. Cells were harvested in 30-min increments. Total RNA extracted from cells was used to synthesize cDNA for qPCR analysis. *GCN4* abundance was normalized to *CHS6* abundance. Data shown are from three biological replicates.

Serial dilution plates showed that *gcn4*Δ grew on all tested sources except for methionine, again sharing this phenotype with *gcn2*Δ (Figure 6A). In addition, spotting *gcn4*Δ onto methionine agar plates supplemented with 10 mM ascorbic acid rescued its growth (Figure 6B). Finally, serial dilution plates with 1-3 mM hydrogen peroxide showed that the *gcn4*Δ strain had impaired growth in the presence of peroxide compared to the wild-type, indicating a sensitivity to oxidative stress, another phenotype shared by the *gcn2*Δ strain (Figure 6C). The *gcn4*Δ:*GCN4* strain showed growth comparable to the wild-type in all conditions assayed. These results implicate Gcn4 in both methionine metabolism and the oxidative stress response in *C. neoformans*.

To ensure that these sensitivities were specific to oxidative stress and not indicative of another stressor such as nitrosative stress sensitivity, cells were spotted onto agar plates containing ammonium as a nitrogen source at pH 4.0, and up to 3 mM of NaNO_2_ was added to each plate. Both the *gcn2*Δ and *gcn4*Δ mutants exhibited wild-type growth in the presence of NaNO_2_, suggesting that Gcn2 and Gcn4 functions are specific to oxidative stress, and do not regulate the response to nitrosative stress in *C. neoformans* (Supplementary Figure 4).

To determine whether Gcn2 has any regulatory control over steady-state *GCN4* mRNA abundance, we examined steady-state levels of *GCN4* via qPCR (Figure 6D). We did not observe any changes in steady-state *GCN4* mRNA abundance in cells incubated in methionine media compared to ammonium media, and we did not observe any difference in abundance between the wild-type and *gcn2*Δ strains. These results indicate that *GCN4* is not regulated at the transcriptional level when the cell is exposed to a methionine-induced oxidative stressor.

We then examined the steady-state abundance of the *ERG110, GST1*, and *GPX1* transcripts in *gcn4*Δ in methionine, to determine whether Gcn4 had any influence over their expression. Cells grown in methionine media showed some increased abundance of *ERG110* compared to cells incubated in ammonium media; however, the increase in abundance was not comparable to what was observed in wild-type or *gcn2*Δ cells, and it was not found to be statistically significant. In addition, incubation in methionine media did not have any effect on the abundances of *GST1* or *GPX1* transcripts in the *gcn4*Δ mutant (Supplementary Figure 5).

## Discussion

*Cryptococcus neoformans* is a fungus under stress. Upon entering the human body, fungal cells encounter stressors such as elevated host temperatures, oxidative stress, changes in pH, and nutrient scarcity (Brown et al., 2007). In order to adapt to the stressors, *C. neoformans* relies on post-transcriptional regulation of gene expression at the levels of both mRNA processing and translation (Bloom et al., 2017). The kinase Gcn2 is essential for the response to oxidative stress in *C. neoformans*, repressing cap-dependent translation through phosphorylation of the initiation factor eIF2. In other fungal species, Gcn2 is essential to the response to amino acid limitation, although a similar role has yet to be characterized in *C. neoformans* (Hinnebusch, 2005; Duncan et al., 2018; Leipheimer et al., 2019a,b). In this study we present evidence indicating a role for eIF2 phosphorylation in regulating methionine metabolism, and we also show that methionine induces an oxidative stress response at the transcriptional and translational levels of gene expression as well as induces a modest accumulation of intracellular ROS. Our work suggests a possible connection between nitrogen metabolism and oxidative stress through the transcription factor Gcn4, potentially indicating the presence of a compound stress response in this clinically significant fungal pathogen.

Little is known about the availability of different nitrogen sources in different niches of the human body, which compounds fungal pathogens utilize, and to what degree such compounds are utilized (Ene et al., 2014). Thus, it is unclear whether there is any environmental niche or situation where methionine would be the only nitrogen source available to *C. neoformans*. However, our study reveals a mechanism by which eIF2 phosphorylation in response to nitrogen limitation also gives rise to an oxidative stress response. A similar phenomenon known as cross-protection has been observed in *Saccharomyces cerevisiae:* the response to heat shock induces antioxidant responses and the cell wall integrity pathway. Culturing cells in hypoxic conditions also results in increased thermotolerance (Morano et al., 2012). In addition, glucose limitation activates the transcription factor Hsf1 that promotes expression of heat shock proteins (Hahn and Thiele, 2004). As *C. neoformans* will encounter multiple stressors simultaneously inside the human host, it is interesting to consider the possibility of compound stress and cross-protection responses and how they contribute to survival inside the human host and pathogenicity.

Methionine has a key role in protein translation as the first amino acid encoded in polypeptide chains, but metabolism of this amino acid is also prominent in many pathways involving cell signaling, proliferation, and survival in eukaryotes (Walvekar and Laxman, 2019). In this study we showed that methionine induces an oxidative stress response in *C. neoformans*. The impaired growth observed in the *gcn2*Δ mutant when methionine was the sole nitrogen source was not induced when methionine was included with ammonium in medium, suggesting that is indeed methionine catabolism and not methionine signaling at the root of the *gcn2*Δ phenotype. Because methionine is indirectly involved in the synthesis of glutathione, we initially speculated that expression of a component of the glutathione synthesis pathway could be regulated by eIF2 phosphorylation. We found that incubation in methionine media induced upregulation of *GPX1* and *GST1* transcripts. However, neither of these proteins are directly connected to glutathione synthesis. In addition, N-acetylcysteine did not rescue growth of *gcn2*Δ cells on methionine (Figure 3A). N-acetylcysteine's antioxidant activity lies greatly in its role as a precursor of reduced glutathione, replenishing glutathione levels whenever they are depleted during oxidative stress, as well as its ability to reduce protein disulfide bonds through its thiol-disulfide exchange activity (Sun, 2010; Aldini et al., 2018). Keeping its role as a glutathione precursor in mind, our results do not suggest a direct connection between eIF2 phosphorylation, methionine catabolism, and glutathione synthesis or oxidant-induced protein disulfide bond formation. More research is needed to determine the exact mechanism connecting methionine to oxidative stress.

After observing that wild-type cells incubated in methionine underwent cap-dependent translation repression, we hypothesized that adding ascorbic acid to cells grown in methionine media would bypass the requirement for an oxidative stress response, leading to growth and translation in methionine similar to that in ammonium. However, we were surprised to find that cells incubated in methionine with ascorbic acid exhibited an even more drastic repression of translation compared to cells incubated in methionine alone (Figure 4B). Since ascorbic acid can act as a pro-oxidant in addition to acting as an antioxidant, we considered that an increase in pro-oxidant activity could contribute to increased translation repression. A reaction between iron and ascorbic acid can generate hydrogen peroxide, increasing oxidative stress and leading to cleavage of the 25s rRNA in the 60s ribosomal subunit (Zinskie et al., 2018; Guth-Metzler et al., 2020; Kazmierczak-Barańska et al., 2020). If this reaction was occurring, it could explain the accumulation at the 40s peaks in the ascorbic acid polysome profiles. However, since both WT and *gcn2*Δ see improved growth when ascorbic acid is added to methionine agar and ascorbic acid alleviates p-eIF2, it is more likely that ascorbic acid's antioxidant effects outweigh its pro-oxidant effects. Due to the improved growth that we observed, we speculate that ascorbic acid's effects on translation protect cells from oxidative stress. Repressing translation during stress allows the cell to conserve energy and resources as it reprograms gene expression to prioritize survival and stress adaptation (Liu and Qian, 2014). Further work will be required to determine the exact mechanisms behind ascorbic acid-mediated translation repression and its role in the oxidative stress response.

First characterized in *S. cerevisiae*, translation of *GCN4* mRNA is a classic example of how a transcript's translation can be differentially regulated in stress conditions. *S. cerevisiae GCN4* contains four upstream open reading frames (uORFs) in its 5' leader, each playing a distinct role in proper translation regulation (Hinnebusch, 2005). During conditions of amino acid starvation, eIF2 phosphorylation promotes enhanced translation of *GCN4* mediated by the uORFs to promote amino acid synthesis (Hinnebusch, 2005). *GCN4* translation can also be increased by hydrogen peroxide stress, and this increase in expression is essential for the peroxide stress response (Mascarenhas et al., 2008). uORF-mediated translation regulation of *GCN4* in response to oxidative stress and eIF2 phosphorylation has also been observed in *Candida albicans*. Gcn4 in *C. albicans* (Sundaram and Grant, 2014a,b) plays a role in regulating morphogenic changes in response to amino acid starvation, an important virulence determinant in this fungal pathogen.

The *GCN4* transcript in *C. neoformans* has two uORFs in its 5' leader, and ribosome profiling data revealed that when the cell is unstressed ribosome occupancy is higher in the uORFs compared to the main coding sequence (Wallace et al., 2020). Our results provide evidence that Gcn4 expression is influenced by Gcn2 and eIF2 phosphorylation, suggesting that *GCN4* translation may be regulated via its uORFs. Our RT-qPCR data provides further evidence that Gcn2's influence on Gcn4 expression is at the translational level, as we saw no changes in steady-state *GCN4* abundance in Gcn2-deficient cells. Examining whether Gcn4 regulated transcription of *ERG110, GST1*, and *GPX1* gave varying results. Although abundances of *GST1* and *GPX1* did not differ between ammonium-incubated and methionine-incubated *gcn4*Δ cells, we observed an increase in *ERG110* abundance in methionine-incubated cells compared to ammonium-incubated cells. These results could indicate a role for Gcn4 in regulating transcription of these genes, yet it does not appear that it is the sole factor responsible for the oxidative stress response in *C. neoformans*. Other transcription factors contribute to the oxidative stress response in *C. neoformans* such as Gsb1 and Atf1. Both of these factors play key roles in peroxide signaling and resistance (Missall and Lodge, 2005; Cheon et al., 2017). It is possible that the transcripts whose abundances we analyzed is regulated by one of these or another transcription factor. Further work will be required to determine the specific transcriptional targets of Gcn4 in relation to ROS stress responses.

The results of our study suggest a connection between nitrogen metabolism and oxidative stress in *C. neoformans*, indicating the presence of a compound stress response that may allow this clinically relevant fungal pathogen to address multiple stressors at a time in order to survive and adapt to the host environment. The connection between these two stressors is mediated by the transcription factor Gcn4, and our work prompts further investigation into how Gcn4 contributes to eIF2-mediated stress responses. The magnitude of stress induction seen in methionine medium is less in magnitude than that seen in response to peroxide, yet the biological consequences of failing to mount an appropriate oxidative response result in a major fitness cost. Future work will investigate how *C. neoformans* has employed the evolutionarily conserved, uORF-mediated regulation of the *GCN4* mRNA to promote stress adaptation, and its potential role in pathogenesis.

## Data Availability Statement

The raw data supporting the conclusions of this article will be made available by the authors, without undue reservation.

## Author Contributions

AS designed and conducted experiments, analyzed data, interpreted the results, and wrote and edited the manuscript. CK contributed to polysome profiling experimental design, conducted polysome profiling, and contributed to data analysis. MK contributed to flow cytometry experimental design, conducted flow cytometry, and performed data analysis. JP contributed to designing experiments, data interpretation and analysis, and manuscript revisions and edits. All authors contributed to the article and approved the submitted version.

## Conflict of Interest

The authors declare that the research was conducted in the absence of any commercial or financial relationships that could be construed as a potential conflict of interest.
