# Supplementary material for: A Conserved Gcn2-Gcn4 Axis Links Methionine Utilization and the Oxidative Stress Response in Cryptococcus neoformans
Source: Front Fungal Biol. 2021 Mar 22;2:640678. doi: 10.3389/ffunb.2021.640678 (PMC8494424; doi:10.3389/ffunb.2021.640678)
Supplement: Supplementary file 1 [file Data_Sheet_1.PDF]

Primer name	Primer sequence
GCN4 CNAG_06246 Forward with 5' Kpn1 restriction site (used for both cloning and screening)	TAATAAGGTACCGGACGAACTGCCTACCTGTG
GCN4 CNAG_06246 Reverse with 5' Kpn1 restriction site (used for both cloning and screening)	TAATAAGGTACCGAATGGAGTGGTGAGCTTGGA
GPX1 CNAG_02503 qPCR Forward	GACTTCAACCACGCAAAGACC
GPX1 CNAG_02503 qPCR Reverse	GGGTCTTGTCGTCCTTGGTT
GST1 CNAG_04110 qPCR Forward	ACCCGCTCGAAAAGAGTCAG
GST1 CNAG_04110 qPCR Reverse	ACGGGCTGTTTCATCTTGGT
ERG110 CNAG_05842 qPCR Forward	GTCGCGACGGAAAACAGATT
ERG110 CNAG_05842 qPCR Reverse	GGACCCCAGATAGCAGGAGA
GCN4 CNAG_06246 qPCR Forward	TGTAGAGATGGAAGTGGCGG
GCN4 CNAG_06246 qPCR Reverse	TGGGACCAGTTTTTGGTGGA
GSH1 CNAG_04647 qPCR Forward	TCCCTTCCCTTGGTCCTCAA
GSH1 CNAG_04647 qPCR Reverse	GAGGTGGTCAGTGAGTTGGG
TRR1 CNAG_05847 qPCR Forward	TCAGAAGAGACGAGCTCCGA
TRR1 CNAG_05847 qPCR Reverse	GTCACCCTTGGCCTCAGTAG
TSA1 CNAG_03482 qPCR Forward	GTCTCAGACCAAGAGGAGCG
TSA1 CNAG_03482 qPCR Reverse	AATGAAGAAGGTGCCTCGGA
CAT1 CNAG_04981 qPCR Forward	GCATTTGAAGCGGCTGGATG
CAT1 CNAG_04981 qPCR Reverse	AAGAAGGTAGTGCGACAGCC
CAT3 CNAG_00575 qPCR Forward	CGTGAACTACACGCTCTGGA
CAT3 CNAG_00575 qPCR Reverse	ACGGACAATTCCCTTCGAGC
CHS6 CNAG_06487 qPCR Forward	TTGACCCTTGGCACATCT
CHS6 CNAG_06487 qPCR Reverse	GTTGGCATAAGTATCCTT

Supplementary Table 1. Primers used in this study.

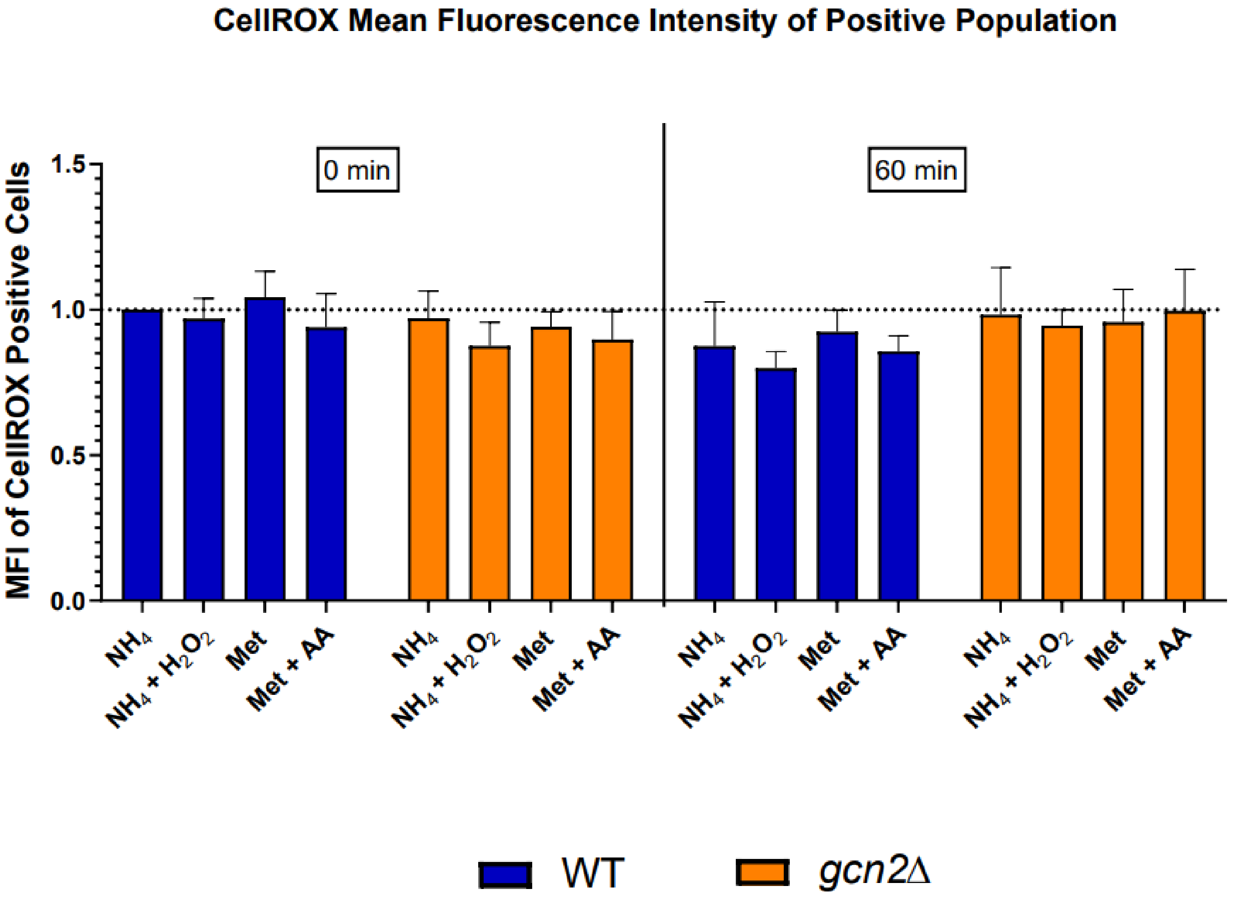

Supplementary Figure 1. Mean fluorescent intensities of CellRox+ cell populations.

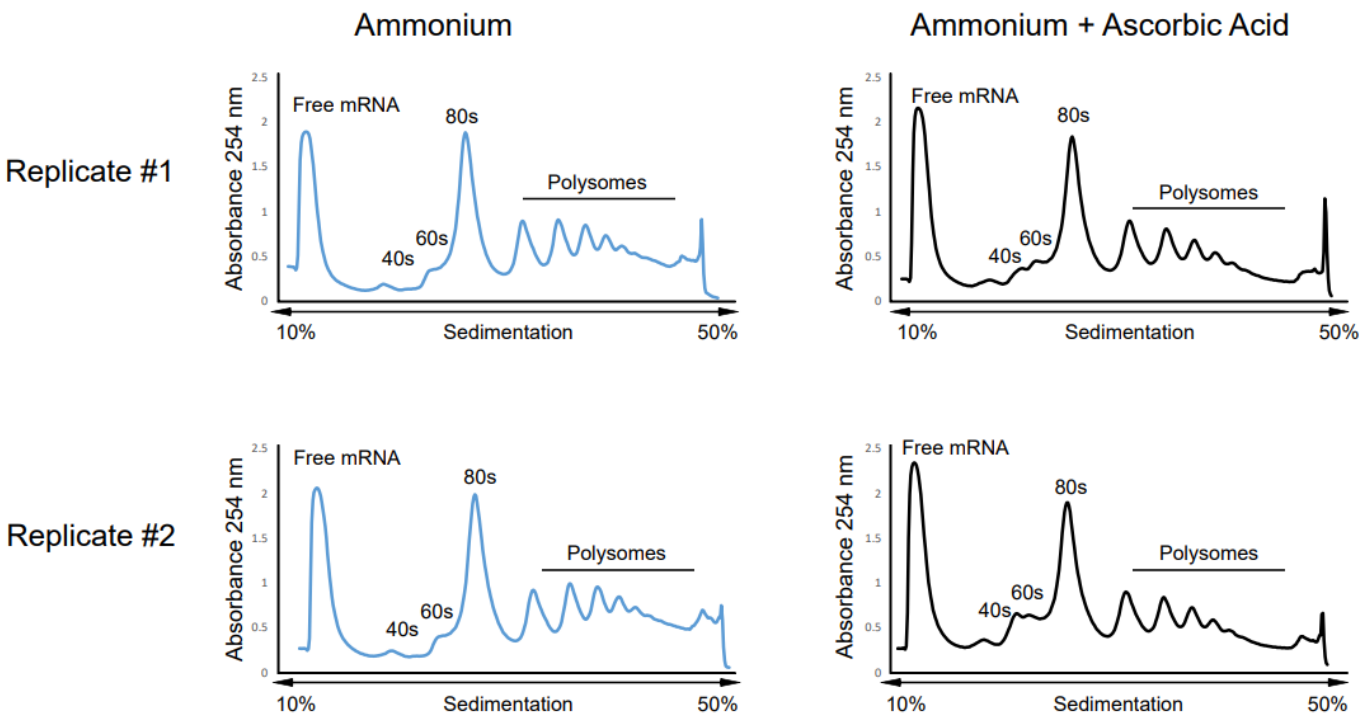

Supplementary Figure 2. Polysome profiling of wild-type cells in ammonium media and ascorbic acid. Mid-log cells were incubated in minimal defined media with ammonium, either supplemented with 10mM ascorbic acid or without ascorbic acid, for 60 minutes. Cells were then harvested and processed for polysome profiling as previously described. Individual biological replicates are shown (replicate #1 on the top row, replicate #2 on the bottom row)

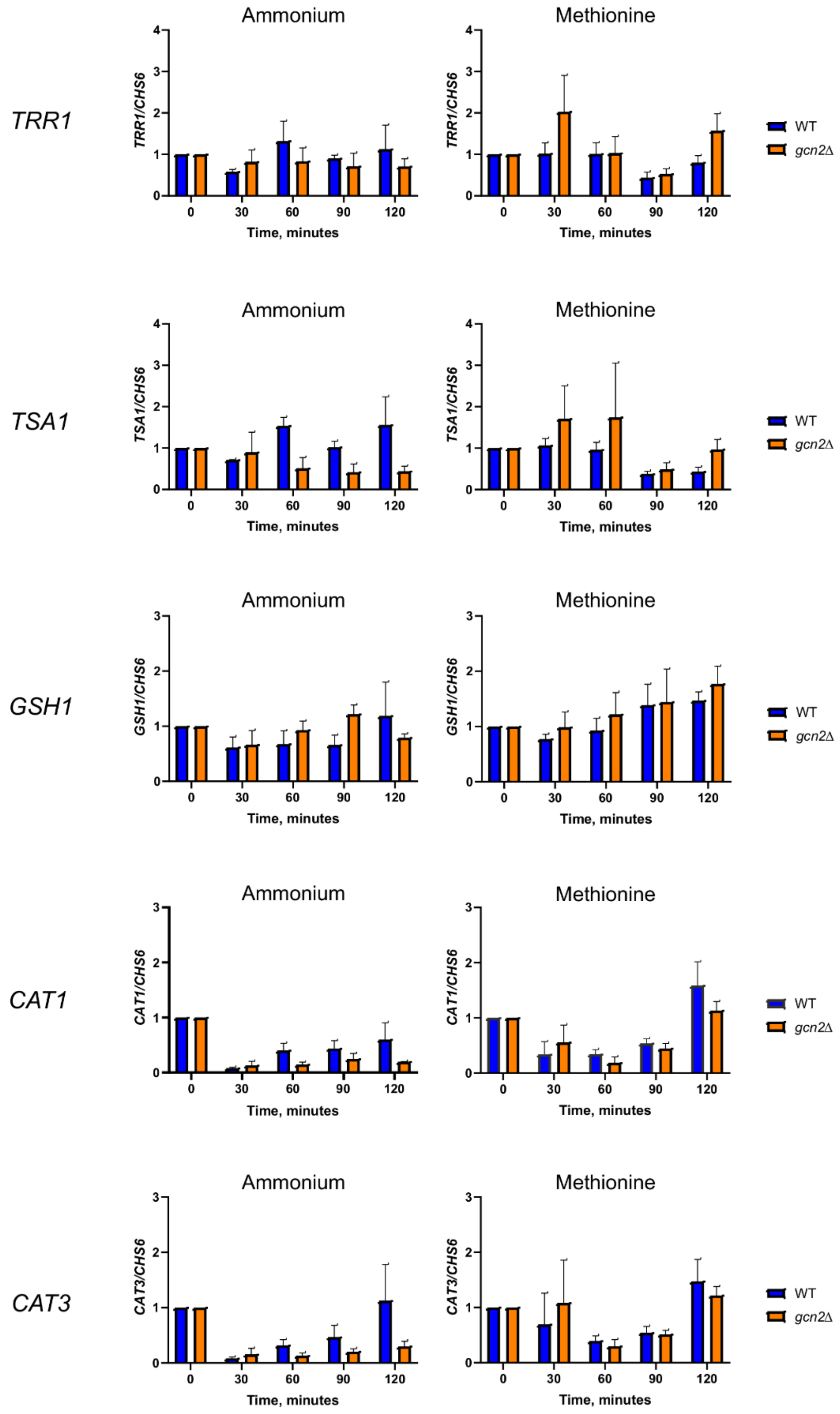

Supplementary Figure 3. RT-qPCR analysis of steady-state abundance of select oxidative stress response transcripts in methionine.

Mid-log wild-type or *gcn2*Δ cells were resuspended in fresh ammonium or methionine media. Cells were collected over a two-hour time course in 30-minute increments. Total RNA extracted from cells was used to synthesize cDNA for qPCR analysis of the abundance of *TRR1*, *TSA1*, *GSH1*, *CAT1*, and *CAT3*. Abundance values for each gene were normalized to *CHS6* values in the same strain at the same timepoint. Data shown are from five biological replicates.

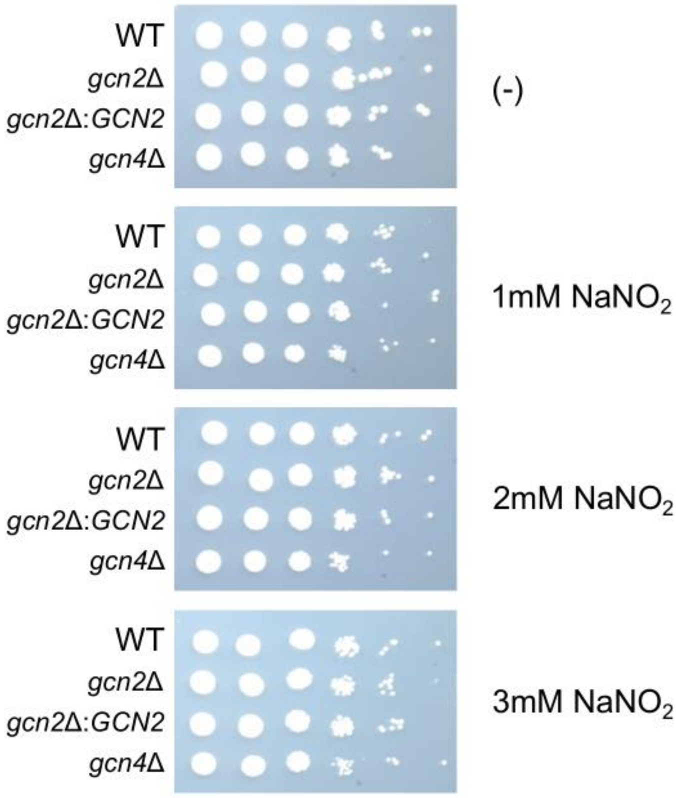

Supplementary Figure 4. Analysis of nitrosative stress sensitivity.
Serial dilution analysis of cells in the presence of different nitrogen sources and antioxidants. Wildtype, *gcn2*Δ, *gcn2*Δ:*GCN2,* and *gcn4*Δ serial dilutions were spotted onto agar plates containing YNB, 2% dextrose with 10mM of ammonium sulfate and sodium nitrite at the indicated concentrations. Plates were incubated at 30° for 2 days before imaging. Images shown are representative of two biological replicates.

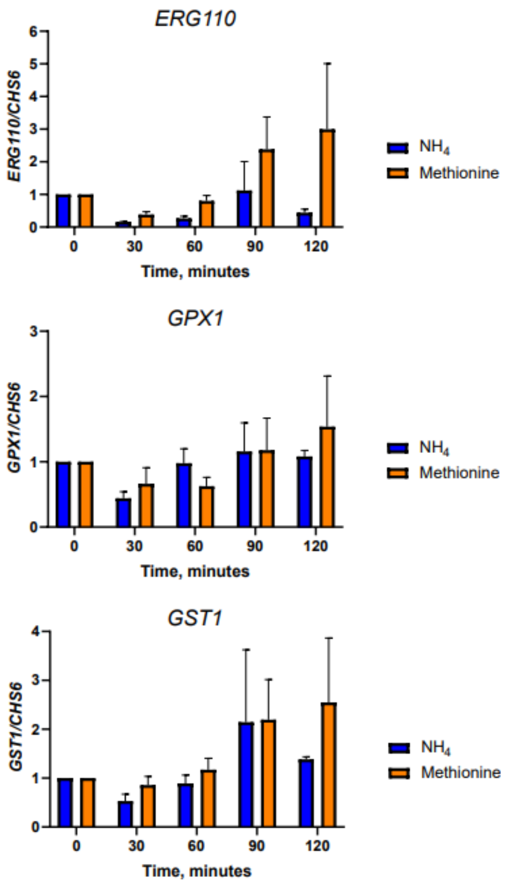

Supplementary Figure 5. RT-qPCR analysis of steady-state abundance of select oxidative stress response transcripts in *gcn4*Δ.
Mid-log *gcn4*Δ cells were resuspended in fresh ammonium or methionine media. Cells were collected over a two-hour time course in 30-minute increments. Total RNA extracted from cells was used to synthesize cDNA for qPCR analysis of the abundance of *ERG110* **(A)**, *GPX1* **(B)**, and *GST1* **(C)**. Abundance values for each gene were normalized to *CHS6* values in the same strain at the same timepoint. Data shown are from five biological replicates.
